# Patient Harm From Orthodontic Care (Part III): The Care Provider Perspective and the Second Victim Phenomenon

**DOI:** 10.1111/ocr.70195

**Published:** 2026-10-11

**Authors:** Kristine Læssøe‐Arendt, Nikolaos Ferlias, Julian Woolley, Peter Stoustrup

**Affiliations:** ^1^ Section of Orthodontics, Department of Dentistry and Oral Health Aarhus University Aarhus Denmark

## Abstract

**Background:**

Understanding the psychological and professional consequences of patient harm events is crucial for improving clinical practice and support systems.

**Aim:**

(1) To investigate the prevalence of self‐reported patient harm events involving orthodontic professionals, (2) to assess the psychological, physical, and professional impact of these events, and (3) to identify aspects of importance to mitigate the psychological and physical impact among orthodontic care providers involved in patient harm events.

**Materials & Methods:**

An online questionnaire‐based survey was conducted among orthodontic care providers, including orthodontists, dentists, hygienists, and dental assistants during two orthodontic conferences in Denmark. The questionnaire assessed respondents' experience with patient harm events, types of harm, the psychological and physical impact of being involved, and their working environment.

**Results:**

A total of 297 participants completed the survey, in which 45.9% (*n* = 135) reported involvement in a patient harm event and 20.8% (*n* = 61) experienced an event within the past year. Following a harmful event, significant psychological distress was reported by 49.2% (*n* = 87) of professionals, while 21.5% (*n* = 38) experienced physical symptoms and 8.8% (*n* = 16) considered leaving their job. A fear of causing harm was also evident, with 32.4% (*n* = 95) participants experiencing high to very high levels of psychological stress. Orthodontists reported greater stress related to the risk of causing harm compared to other orthodontic team members (*p* = 0.001). Orthodontists involved in a patient harm event also reported greater psychological (*p* = 0.002) and physical impact (*p* = 0.001) than other clinical staff members. No significant differences were found based on age, gender or years of clinical experience. Respondents most frequently identified structured organizational support, supportive leadership, and peer support as key measures to mitigate the impact of patient harm events.

**Conclusion:**

Patient harm events may affect orthodontists and staff and potentially cause significant psychological and physical impact, emphasizing the need for structured support and peer programmes to enhance provider well‐being and patient safety.

## 1 Introduction

Historically, advancements in healthcare systems have often focused on improving quality of care, with patient safety receiving increasing attention over time [1]. According to the World Health Organization (WHO), patient safety is defined as ‘the absence of preventable harm to a patient and reduction of risk of unnecessary harm associated with health care to an acceptable minimum’ [2]. The goal of patient safety is to minimize preventable harm, accidents, and mitigate adverse events (AEs) and patient safety events [2, 3].

In dentistry and orthodontics there is a significant lack of research regarding patient safety [4]. Dental and orthodontic treatments are often highly technical procedures performed in complex environments, which inherently increases the risk of errors and patient harm [5, 6, 7]. Despite the growing awareness of patient safety in other areas of healthcare, little attention has been given to the psychological and physical impact of adverse events on dental professionals [8, 9].

The ‘Second Victim Phenomenon’ is a well‐established concept in broader medical settings, describing the emotional distress experienced by healthcare professionals involved in patient safety events, medical error, patient harm, or adverse events [10, 11, 12, 13]. The phenomenon was first described by Albert W. Wu in 2000, in which clinicians involved in adverse events often suffer significant psychological and professional distress, termed ‘second victims’ [10]. While traditional patient safety efforts primarily focus on the affected patients, increasing evidence demonstrates consequences for the healthcare providers involved [14]. These professionals may experience physical and psychological distress, burnout, reduced job satisfaction, and, in severe cases, contemplate leaving their profession [4, 5, 6].

Addressing the issue the ‘Second Victim Phenomenon’ within the field of orthodontics is crucial for fostering a resilient workforce and enhancing the quality of patient care; ultimately enhancing patient safety. Given the limited evidence regarding the impact of patient harm events on orthodontic care providers, this study aimed to (1) investigate the prevalence of patient harm events among them, (2) quantify their psychological, physical, and professional consequences, and (3) identify workplace factors perceived to be important for supporting orthodontic care providers following such events. By doing so, the study seeks to provide an epidemiological overview of the second victim phenomenon within orthodontic practice.

## 2 Materials and Methods

The present study is a questionnaire‐based investigation. Participants were orthodontic care providers attending two orthodontic conferences, one held in Randers, Denmark, on January 21, 2024, and subsequently in Vejle, Denmark, on September 20, 2024. Recruitment was carried out by two of the authors (KLA and PS). The term ‘orthodontic care providers’ refers collectively to orthodontists, dentists, dental hygienists, and clinical assistants involved in orthodontic care. All attendees present during the survey sessions were invited to participate and no incentives were offered. Participation was voluntary, and as the total number of eligible attendees was unknown, a response rate could not be calculated. However, participants represented the majority of orthodontic care providers in Denmark. The nature of the questionnaire was also explained and it was made clear that participation in the survey was entirely voluntary. The questionnaire was completed in private using the online questionnaire tool Mentimeter (Stockholm, Sweden).

In compliance with Danish regulations on questionnaire‐based research, ethical approval was not deemed necessary for this study. All data were collected anonymously and all participants provided written consent for the use of their responses. It also adhered to the terminology previously proposed for orthodontic‐related patient‐harm [4].

### 2.1 Questionnaire

Prior to the first conference, a standardized questionnaire was developed to explore the multidimensional aspects of patient harm and its potential impact on orthodontic care providers. The questionnaire was developed specifically for the aims of the present study using the validated Second Victim Experience and Support Tool (SVEST) as the primary framework for questionnaire construction [15]. Selected items from the SVEST were adapted to reflect the orthodontic clinical setting, while additional items were developed by the authors to assess orthodontic‐specific patient harm events, workplace practices, and support needs that were not covered by the original instrument.

The questionnaire used in this study comprised 22 questions addressing various dimensions of patient harm and patient safety in a Likert‐Scale format (Table 1). Participants were invited to complete the questionnaire via the online Mentimeter (Stockholm, Sweden) tool during the conferences. The survey included four demographic questions about age, gender, title, and level of experience (Q1–Q4), followed by four questions concerning their experiences with patient harm and the resulting impact (Q5–Q8). An open‐ended free‐text question (Q9) was also included, asking participants to describe patient‐related harm(s) from orthodontic care that they had been involved in. Additionally, the survey featured eight questions (Q10–Q17) examining the psychological and physiological impact of being involved in patient harm events on the orthodontic team members. In addition, the questionnaire included five questions (Q18–Q22) focusing on patient safety practices and the management of such events in the workplace. The last item (Q22) of the survey was an open‐ended question where participants could respond with free text.

**TABLE 1:** Questionnaire used in this study with both Likert‐Scale questions as well as free‐text. Participants responded using an online tool (*n =* 297).

Question number	Question	Response options
Q1	Gender	(1) Male, (2) Female, (3) Do not wish to answer
Q2	Educational background	(1) Orthodontist, (2) Dentist, (3) Dental hygienist, (4) Clinical assistant, (5) Other
Q3	Age category of participant	(1) 18–25years, (2) 26–35years, (3) 36–45years, (4) 46–55year, (5) > 55year
Q4	Years since graduation	(1) 0–5, (2) 6–15, (3) 16–25, (4) > 25
Experience with patient harm		
Q5	I have been involved in an orthodontic treatment where a patient was harmed	(1) Yes, (2) No, (3) Do not known
Q6	I have been involved in an orthodontic treatment where a patient was harmed within the last 12 months	(1) Yes, (2) No, (3) Do not known
Q7	I am generally nervous about harming a patient during treatment	(0) Not at all, (1) Minor degree, (2) Some degree, (3) High degree, (4) Very high degree
Q8	The risk of harming a patient is a general stressor in my orthodontic practice.	(0) Not at all, (1) Minor degree, (2) Some degree, (3) High degree, (4) Very high degree
Types of harm		
Q9	Specify the type(s) of harm you have been involved in (multiple harms allowed)	Open‐ended free text
Emotional and physical impact of being involved in patient‐harm		
Q10	I felt ashamed of being involved in the incident/injury.	(0) Not been involved in harm, (1) Not at all, (2) Minor degree, (3) Some degree, (4) High degree, (5) Very high degree
Q11	I feared sharing the incident with others.	(0) Not been involved in harm, (1) Not at all, (2) Minor degree, (3) Some degree, (4) High degree, (5) Very high degree
Q12	I was nervous the patient might file a complaint about the incident	(0) Not been involved in harm, (1) Not at all, (2) Minor degree, (3) Some degree, (4) High degree, (5) Very high degree
Q13	The patient injury impacted me mentally afterward (e.g., anxiety, sadness, depression).	(0) Not been involved in harm, (1) Not at all, (2) Minor degree, (3) Some degree, (4) High degree, (5) Very high degree
Q14	The patient injury affected me physically afterward (e.g., appetite, sleep)	(0) Not been involved in harm, (1) Not at all, (2) Minor degree, (3) Some degree, (4) High degree, (5) Very high degree
Q15	The harm made me consider leaving patient care afterward.	(0) Not been involved in harm, (1) Not at all, (2) Minor degree, (3) Some degree, (4) High degree, (5) Very high degree
Q16	I am unsure if I can handle patient/parent situations well if a future harm occurs	(0) Not been involved in harm, (1) Not at all, (2) Minor degree, (3) Some degree, (4) High degree, (5) Very high degree
Q17	I am unsure if I can advise patients/parents on legal matters if a harm occurs.	(0) Not been involved in harm, (1) Not at all, (2) Minor degree, (3) Some degree, (4) High degree, (5) Very high degree
Working environment and harm		
Q18	I feel safe sharing concerns and mistakes without fearing negative reactions from colleagues/leaders (Psychological safety)	(0) Not been involved in harm, (1) Not at all, (2) Minor degree, (3) Some degree, (4) High degree, (5) Very high degree
Q19	We routinely follow up on patient injuries or errors to learn and adjust our practices.	(0) Not been involved in harm, (1) Not at all, (2) Minor degree, (3) Some degree, (4) High degree, (5) Very high degree
Q20	We continually focus on improving patient safety at my clinic.	(0) Not been involved in harm, (1) Not at all, (2) Minor degree, (3) Some degree, (4) High degree, (5) Very high degree
Q21	My workplace has adjusted workflows based on learning from errors/injuries in the past 12 months	(0) Not been involved in harm, (1) Not at all, (2) Minor degree, (3) Some degree, (4) High degree, (5) Very high degree
Q22	What support would help most if involved in a severe patient harm?	Open‐ended free text

Before completing the questionnaire, all participants were provided with a standardized definition of orthodontic‐related patient harm based on the terminology previously proposed by Ferlias et al. [4]. The definition was presented to ensure a common understanding of the term ‘patient harm’ across all participant groups, irrespective of professional background or level of clinical experience. Participants were instructed to answer all subsequent questions using this definition as the reference.

### 2.2 Quantitative Data Analysis

Descriptive statistics were used to summarize and present the quantitative data. The percentages for each harm category were calculated using the total number of respondents as the denominator: Number of participants reporting the harm category divided by the total number of respondents. Participants could report multiple types of patient harm; therefore, the total number of reported harms could exceed the number of respondents who had experienced a patient harm event.

Chi‐square tests and Fisher's exact tests were applied for inter‐group comparisons in the sub‐analyses, using Stata18 software (Texas, USA) with significance level set to *p* = 0.05. Given the exploratory nature of the study, analyses were primarily descriptive and hypothesis‐generating. Associations between variables were assessed using Chi‐square and Fisher's exact tests.

### 2.3 Qualitative Data Analysis

The qualitative data derived from the two open‐ended free‐text questions (Q9 and Q21) were evaluated as follows:

For Q9: ‘Specify the type(s) of harm you have been involved in (multiple harms allowed)’, the free‐text responses were categorized into main harm categories (local and systemic) and subsequent subgroups based on a previously proposed classification system [4]:

Local types of patient harms:

1. Dental tissues (e.g., root resorption, pulp necrosis, caries).
2. Soft tissues (e.g., gingival recessions, soft tissue laceration).
3. Impact on orofacial function.
4. Unwanted tooth movement (e.g., active retainer with excessive incisor torque).

Systemic types of patient harms:

1. Pain and discomfort (e.g., hypersensitivity due to excessive interproximal reduction).
2. Emotional harm (e.g., development of odontophobia).
3. Other adverse events and types of harm (e.g., inhalation of foreign bodies, wrong‐tooth extraction).

Responses to Q22 (‘What support would help most if involved in a severe patient harm?’) were analysed using a large language model (LLM)‐assisted semantic clustering approach. The anonymized free‐text responses were exported to Microsoft Excel (Redmond, WA, USA) and subsequently analysed using ChatGPT 4.0 (OpenAI, San Francisco, CA, USA). A predefined one‐shot prompt instructed the model to identify semantically similar responses and group them into coherent thematic clusters without altering the original content of the responses. Iterative prompts were subsequently used only to refine the organization and presentation of the identified themes. The generated clusters were reviewed by the authors to ensure that they accurately reflected the original participant responses before being reported. Details of the complete prompt are provided in Table S1.

## 3 Results

A total of 297 participants completed the online survey at one of the two occasions. The majority of participants were female (*n* = 254, 85.8%). Of the participants, 43.5% were specialists in orthodontics (*n* = 128), 35.1% (*n* = 103) were clinical assistants, 12.6% (*n* = 37) hygienists and 5.8% (*n* = 17) were dentists without orthodontic specialization. The majority of participants had at least 6 years of orthodontic experience (82%, *n* = 240) (Table 2).

**TABLE 2:** Demographic description of participants with educational background and years of experience (*n =* 297).

Question	Response
Q1: Gender	Male: 13.51% (*n* = 40) Female 85.81% (*n* = 254) No info: 0.68% (*n* = 2)
Q2: Educational background	Orthodontist: 43.54% (*n* = 128) Dentist: 5.78% (*n* = 17) Dental hygienist: 12.59% (*n* = 37) Clinical assistant: 35.03% (*n* = 103) Others: 3.06% (*n* = 9)
Q3: Age category of participant	18–25years: 0% (*n* = 0) 26–35years: 16.26% (*n* = 47) 36–45years: 33.22% (*n* = 96) 46–55years: 29.07% (*n* = 84) > 55years: 21.45% (*n* = 62)
Q4: Years since graduation	0–5years: 18.09% (*n* = 53) 6–15years: 36.86% (*n* = 108) 16–25years: 24.57% (*n* = 72) > 25years: 20.48% (*n* = 60)

### 3.1 Experience With Patient Harm

Table 3 shows that 45.9% (*n* = 135) of participants reported involvement in a patient harm event related to orthodontic treatment during their career, while 20.8% (*n* = 61) reported involvement in a patient harm event within the past 12 months. When asked about the risk of harming a patient during treatment, 32.4% of participants reported being nervous about harming a patient to a ‘high degree’ or ‘very high degree’, compared to 4.4% who were ‘not at all’ nervous. When asked about the risk of harmful events, 18.1% of participants indicated that the risk of harming a patient was a ‘high’ or ‘very high’ stressor, while 10.2% stated they were ‘not at all’ affected by this risk.

**TABLE 3:** Experience with patient harm.

Question	
Q5: I have been involved in an orthodontic treatment where a patient was harmed	Yes: 135 (45.92%) No: 136 (46.26%) Do not know: 23 (7.82%)
Q6: I have been involved in an orthodontic treatment where a patient was harmed within the last 12 months	Yes: 61 (20.82%) No: 207 (70.65%) Do not know: 25 (8.53%)
Q7: I am generally nervous about harming a patient during treatment	Not at all: 13 (4.44%) Minor degree: 91 (31.06%) Some degree: 94 (32.08%) High degree: 64 (21.84%) Very high degree: 31 (10.58%)
Q8: The risk of harming a patient is a general stressor in my orthodontic practice.	Not at all: 30 (10.24%) Minor degree: 109 (37.20%) Some degree: 101 (34.47%) High degree: 39 (13.31%) Very high degree: 14 (4.78%)

Figure 1 shows the classification of harmful events reported by the participants. The most frequently reported harm types were severe root resorptions (*n* = 185), followed by harm due to delayed or missing diagnostics (*n* = 68).

**FIGURE 1:**
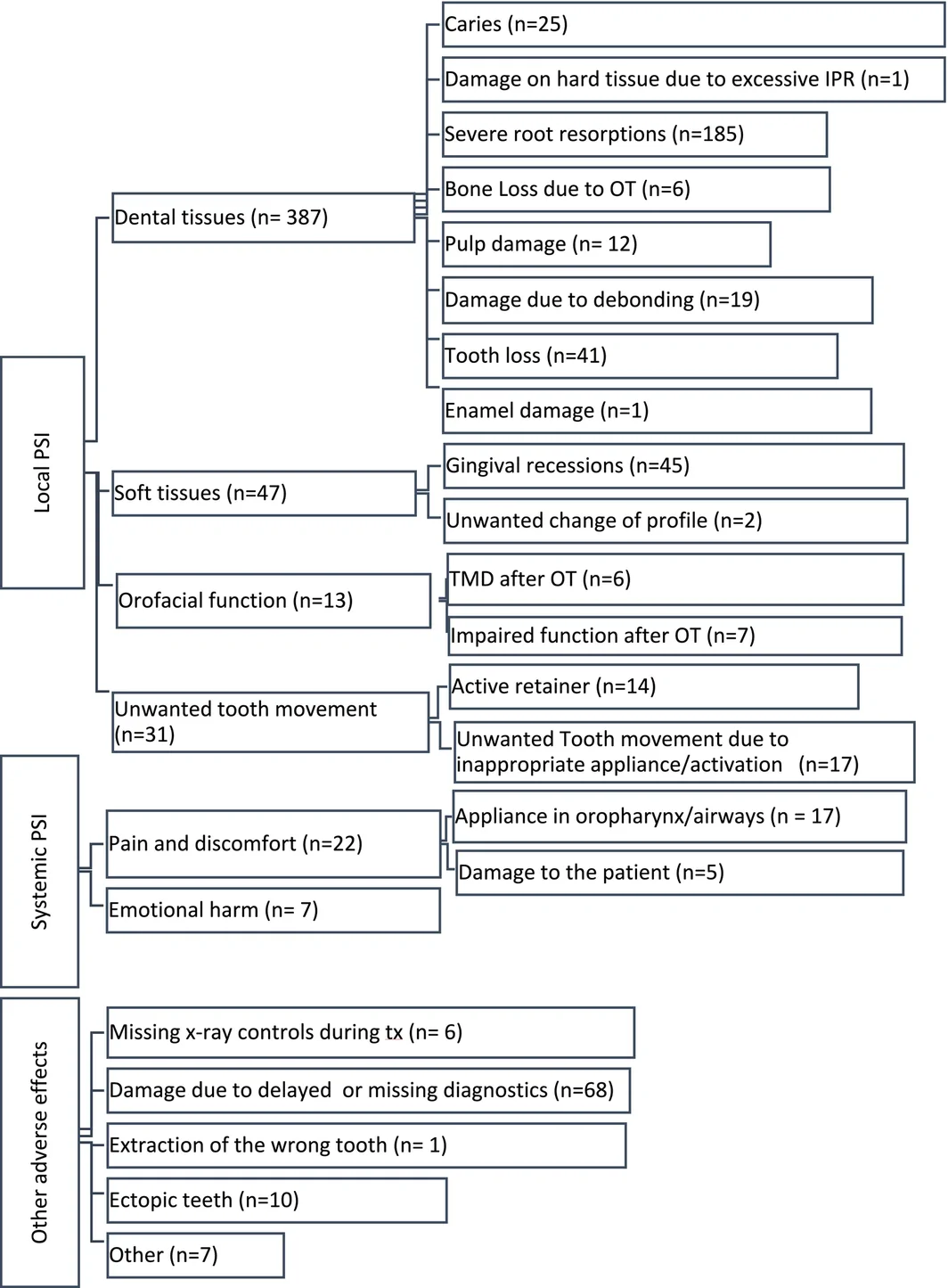
Different types of harm as reported by the participants who were involved. Most participants were involved in more than one patient harm incident. Classification based on the proposed model by Ferlias et al., 2024 (4). OT, Orthodontic treatment; TMD, Temporomandibular Disorder; Tx, Treatment.

### 3.2 Physiological and Physical Impact of Being Involved in Patient Harm

Figure 2 demonstrates the physiological and physical impact of being involved in patient harm, with many participants reporting notable impact across multiple dimensions. For Q10 (‘I felt ashamed of being involved in the event/injury’), 61.6% of participants (*n* = 109) reported feeling ashamed of being involved in the patient safety event to ‘some degree’ or higher, compared to 14.7% (*n* = 26) who reported no feelings of shame. Similarly, in Q13 (‘The patient injury impacted me mentally afterward (e.g., anxiety, sadness, depression)’), 49.1% (*n* = 87) stated that the patient injury impacted them mentally to ‘some degree’ or higher, while 19.2% (*n* = 34) reported no mental impact. In Q14 (‘The patient injury affected me physically afterward (e.g., appetite, sleep)’) 21.5% (*n* = 38) presented these physical effects to ‘some degree’ or higher, compared to 54.2% (*n* = 96) who experienced no impact. Regarding Q15 (‘The harm made me consider leaving patient care afterwards’) 8.8% (*n* = 16) considered leaving patient care to ‘some degree’ or higher, whereas 76.2% (*n* = 138) reported no such considerations.

**FIGURE 2:**
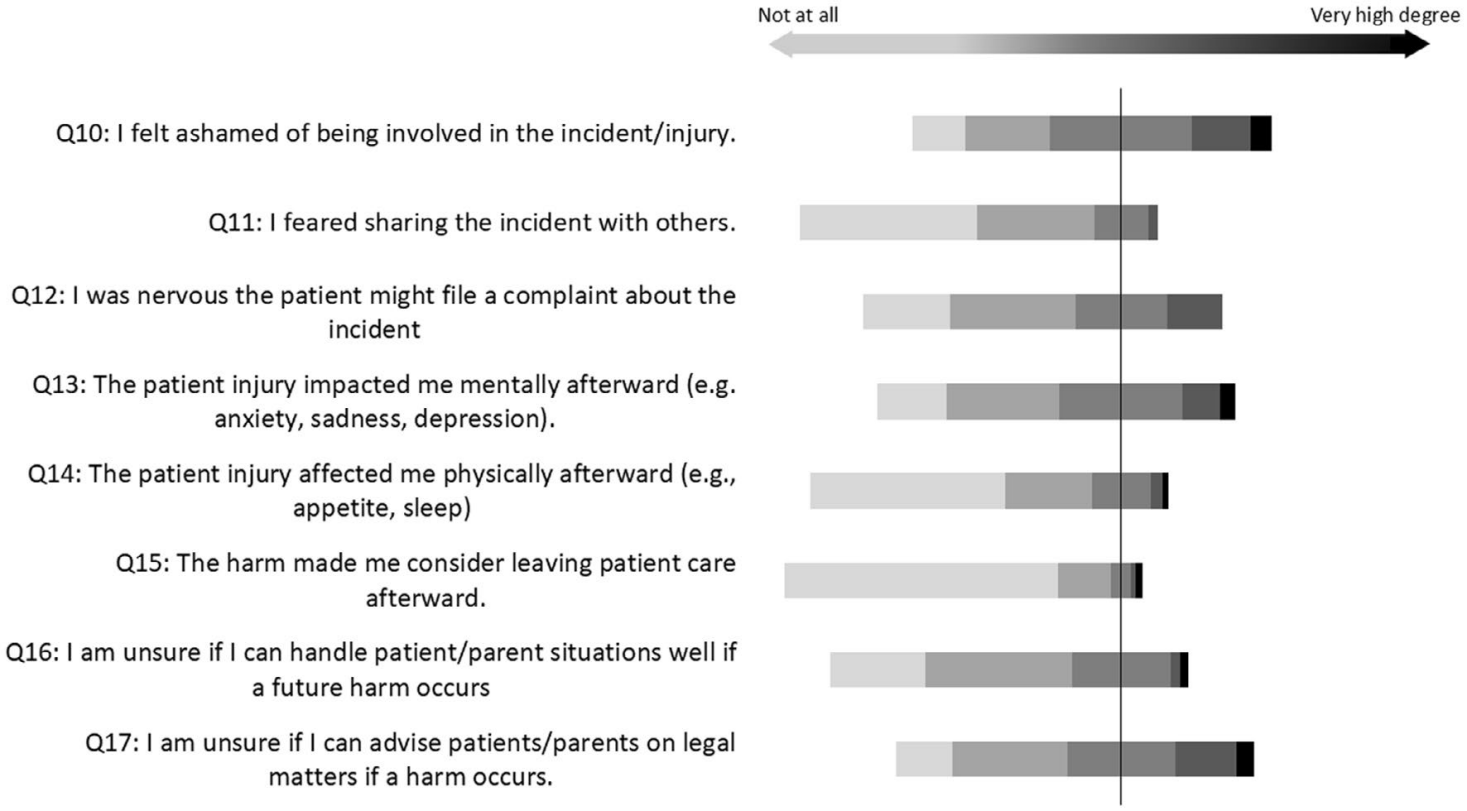
Emotional, physiological and physical impact of being involved in patient‐harm as reported by the participants in items Q10–Q17 with a Likert‐scale format, 0: Not at all, 1: Minor degree, 2: Some degree, 3: High degree, 4: Very high degree. Vertical line represents the scale midpoint (score 2).

### 3.3 Influence of Educational Background, Age, Gender, and Years Since Completion of Final Educational Degree

Subgroup analyses of Q7–Q17 assessed the impact of education, age, gender, and years since the final degree. These factors were examined for their effect on psychological and physical outcomes. Orthodontists reported a significantly higher degree of stress related to the risk of causing harm compared to other members of the orthodontic team (Q8, *p* = 0.001). Additionally, orthodontists who had been involved in a patient harm event reported significantly greater psychological impact (Q13, *p* = 0.002) and physical impact (Q14, *p* = 0.001) than their clinical staff counterparts who had been involved in a patient harm event. Factors such as age, gender, and years since graduation did not have a significant influence on the reported outcomes.

### 3.4 Working Environment and Harm

Most participants felt psychologically safe sharing concerns with 79.5% (*n* = 221) reporting ‘high’ or ‘very high’ degree of psychological safety in Q18 (‘I feel safe sharing concerns and mistakes without fearing negative reactions from colleagues/leaders (Psychological safety)’). Furthermore, 73.4% (*n* = 204) of the respondents indicated that workplaces routinely follow up on patient injuries to improve practices to Q19 (‘We routinely follow up on patient injuries or errors to learn and adjust our practices’). Additionally, 58.5% (*n* = 162) reported a strong focus on improving patient safety (Q20: ‘We continually focus on improving patient safety at my clinic’). However, only 30.2% (*n* = 83) noted workflow adjustments based on learnings from past harm events, while 46.2% (*n* = 127) were uncertain (Q21: ‘My workplace has adjusted workflows based on learning from errors/injuries in the past 12 months’).

### 3.5 Cluster Analysis on Optimal Support When Involved in Harm Events

Table 4 documents the GAI‐generated semantic cluster analysis based on the responses (*n* = 238). The LLM analysis identified five distinct themes regarding support related to patient harm events. Cluster 5, emphasizing the critical role of operational structure, legal clarity and proactive leadership in resolution and prevention of such issues, dominated the responses (52.1%, *n* = 124). Clusters 1 (16.4%, *n* = 39) and 4 (12.6%, *n* = 30) focused on interpersonal and managerial support but differed in emphasis. Cluster 1 demonstrated the need for emotional support from colleagues and leadership right after the patient harm events, while Cluster 4 emphasized a more general proactive leadership and encouragement in creating a supportive and psychologically safe work environment. Finally, Clusters 2 (11.8%, *n* = 28) and 3 (7.1%, *n* = 17) stressed collaborations but from different angles. Cluster 2 emphasized professional sparring and shared problem‐solving immediately after the patient harm event, whereas Cluster 3 focused on teamwork and collegial relationships for addressing challenges with a more general perspective.

**TABLE 4:** Tables of clusters based on the open‐ended questionnaire item Q21: ‘What support would help most if involved in a severe patient harm?’ Data were clustered using artificial intelligence (ChatGPT v4.0).

Cluster	Cluster theme	Total responses	Percentage	Explanation
1	‘Support from colleagues, leadership, and unions’	39	16.39	Emphasizes the importance of emotional and professional backing from colleagues, leadership, and unions to help navigate stressful situations. This cluster highlights the need for empathy and encouragement in stressful situations
2	‘Sparring with colleagues and collaborative discussions’	28	11.76	Highlights the role of peer‐to‐peer interaction and professional sparring in sharing insights, for continuous improvement of practices, and solving problems collaboratively
3	‘Collegial support and teamwork in addressing challenges’	17	7.14	Focuses on the value of strong collegial relationships and team‐based support for effectively addressing and resolving challenges when they occur
4	‘Leadership and collegial encouragement in difficult situations’	30	12.61	Stresses the need for proactive leadership and peer encouragement to foster a positive and supportive work environment during challenging circumstances
5	‘Standardized procedures, legal understanding, and strong leadership’	124	52.1	Highlights the critical role of clear and structured operational procedures, legal clarity, and proactive leadership in ensuring efficient resolution and prevention of future issues

## 4 Discussion

This study is the first to investigate the second victim phenomenon among orthodontic care providers. Overall, the findings largely fulfilled the study aims by providing preliminary data on the occurrence of patient harm events, demonstrating their psychological and physical impact on orthodontic care providers, and identifying workplace support measures considered important following such events. The self‐reported occurrence of patient harm events provides a complementary perspective to the more objective claims‐based data on patient harm in orthodontics reported in Part I of this series [16]. However, the findings relating to the occurrence of patient harm events and preferred support strategies should be interpreted in light of the limitations of the study. Nevertheless, the relevance of the second victim phenomenon is evident with the frequency with which respondents reported exposure to patient harm.

Nearly half of the participants reported having been involved in at least one patient harm event during their clinical careers. Although this finding suggests that patient harm events are not uncommon in orthodontic practice, the estimate should be interpreted with caution because lifetime self‐reported measures are inherently susceptible to recall bias. The responses relating to events occurring within the previous 12 months may provide a more robust estimate of recent exposure. This finding is consistent with the limited orthodontic literature that is available on the subject; previous studies have shown that a substantial proportion of orthodontists experience accidental clinical events at least once during their careers, including soft‐tissue trauma, appliance ingestion, and other unintended events [4, 5, 17, 18, 19, 20]. Analyses of dental patient safety data similarly suggest that orthodontic‐related events represent a recurring component of reported harms, despite acknowledged under‐reporting and variability in definitions [4].

The findings from this study indicate that patient harm events have substantial psychological and physical effect on care providers. Over 61% of respondents reported experiencing shame to some degree, while nearly 50% faced mental health challenges such as anxiety or sadness to some degree. Additionally, 21% noted physical effects, such as disrupted sleep or changes in appetite to some degree. These signs and symptoms are characteristic of the second victim phenomenon, which encompasses a range of emotional (e.g., guilt, anxiety, loss of confidence), cognitive, and physical responses following involvement in an adverse event [21]. Comparable studies in broader healthcare settings have similarly documented high rates of stress among clinicians exposed to safety events, where large surveys of physicians have found that a majority report emotional distress, anxiety, or sleep disturbance after involvement in patient safety events, with some experiencing lasting psychological impact [22]. These emotional and physical reactions have been shown to impair professional functioning and, when unaddressed, may contribute to burnout, defensive clinical behaviour, and considerations of career change [13, 23, 24, 25].

Furthermore, orthodontists reported higher stress levels than other orthodontic care provider groups, likely reflecting the different responsibilities and clinical risks inherent in the job role of an orthodontist. The results from this study also suggest that the role of an orthodontist is a significant factor, but not age, gender, or years of experience [26]. From a human‐factors perspective, this may be partly explained by cognitive processes involved in clinical decision‐making. Dual‐process theory, as described by Kahneman, highlights the interplay between intuitive and analytical thinking, with the latter aligning closely with the structured, protocol‐driven nature of orthodontic practice [27]. However, analytical reasoning under conditions of stress and cognitive load remains vulnerable to bias. Cognitive factors have been implicated in a substantial proportion of diagnostic errors in healthcare, with systematic reviews suggesting that such biases may contribute to errors in 36.5%–77% of cases [28]. These findings underscore the importance of identifying cognitive stressors and human factors in orthodontic decision‐making and patient safety.

Psychological safety is the extent to which people feel their work environment allows them to take interpersonal risks, like speaking up or asking for help, without fear of negative consequences [29]. Although participants reported a high level of psychological safety, 61% of the participants still experienced feelings of shame in relation to patient harm. This aligns with the observation that clinicians involved in errors often require support to cope effectively [10]. In the absence of structured support systems, care providers may adopt maladaptive coping strategies, which can initiate a ‘new chain of victims’ and ultimately compromise patient safety [11, 25, 30]. This means that unaddressed distress in one clinician can propagate harm to others. Moreover, only 30% of respondents indicated that workflows were adjusted following patient harm events, highlighting a gap in translating lessons from adverse events into preventive measures. Implementation of structured support programmes has been shown to provide rapid and accessible assistance for second victims, facilitating recovery and resilience [14, 31]. Addressing these gaps could therefore enhance learning, improve psychological safety, and foster a culture of continuous quality improvement within the orthodontic practice.

### 4.1 Limitations and Strengths

The current study comes with some limitations that need consideration. First, as with all studies relying on self‐reported data, responses may be influenced by recall bias, feelings of shame or guilt, or social desirability, particularly given the sensitive nature of the topic. Also, the cross‐sectional design of the study most likely precludes conclusions regarding causality. Additionally, cultural differences and variations in healthcare systems across countries may limit the generalizability of these findings to orthodontic practice in other settings and countries. Furthermore, the inability of the respondents to differentiate between side‐effect and patient harm events may have influenced the results. Moreover, although the questionnaire was based on a previously validated instrument, several items were modified or newly developed for the orthodontic setting. As the aim was exploratory rather than psychometric, the adapted questionnaire was not formally validated, and the findings should therefore be interpreted accordingly. The questionnaire was administered in Danish, as participants attended Danish orthodontic conferences and routinely communicate professionally in Danish. Since it was adapted for the specific study context rather than intended as a formal Danish version of the SVEST, a forward‐backward translation procedure was not performed. The questionnaire was used primarily to obtain descriptive information on orthodontic care providers' experiences of patient harm events and their perceived psychological and professional impact. Finally, the semantic clustering of free‐text responses was performed using an LLM‐assisted approach. The clustering was qualitatively confirmed by reviewing all free‐text responses. Although this provided an efficient and transparent method for organizing participant responses, neither formal quantitative validation nor assessment of inter‐rater agreement was undertaken. Consequently, these findings should be regarded as exploratory and hypothesis‐generating. The complete prompt has been provided in the Supporting Information to enhance reproducibility.

Important strengths of the current study are: (1) the novelty of the research approach as this is the first study to investigate the second victim phenomenon in the field of orthodontics, (2) the mixed‐methods nature of the study. Due to the collection of both quantitative and qualitative data, a more comprehensive and multidimensional understanding of patient harm experiences was achieved, (3) the real‐world conference‐based recruitment of all participants and their high professional diversity which allowed for subgroup analyses and finally (4) the practical recommendations and actionable opportunities that can be derived from the results in everyday practice.

### 4.2 Clinical Applicability

The clinical applicability of our findings is that they highlight the significant psychological and physical impact of patient harm events on orthodontic care providers, emphasizing the need for structured support systems to promote resilience and prevent burnout. By implementing workflow adjustments, event follow‐ups, and targeted support, clinics can reduce the risk of subsequent errors and improve patient safety. Role‐specific interventions for orthodontists and other team members can further ensure support is tailored to clinical responsibilities and decision‐making demands.

Future research could focus on assessing the long‐term outcomes of patient harm events and evaluating the effectiveness of interventions aimed at reducing the ‘second victim’ phenomenon. Such efforts could align with the World Health Organization's research cycle, contributing to the development of strategies that promote safer care practices in orthodontics [32, 33]. Specifically, future studies could examine the effectiveness of structured support programmes, peer support systems, and leadership training in reducing the emotional and physical burden on clinicians and staff [14, 34, 35]. Research should also explore how systematic event follow‐up, workflow adjustments, and organizational learning can both support affected team members and enhance overall patient safety within orthodontic practice. Finally, future research should also assess the emotional impact of patients being involved in patient safety events as this has not been elucidated in the scientific orthodontic literature yet.

## 5 Conclusion

This exploratory study demonstrates that the second victim phenomenon may affect orthodontists and other orthodontic care providers, with involvement in patient harm events being associated with notable psychological and physical impact. Orthodontists reported higher levels of stress than other team members, which may indicate the role of clinical responsibility in vulnerability to these effects. Although psychological safety was generally reported as high, only a minority of workplaces reported adjusting workflows following patient harm events, indicating a gap in translating lessons into preventive practices. Structured support programmes, peer support, and leadership engagement, which have been described as potentially beneficial in healthcare settings, could enhance provider well‐being and patient safety in orthodontics.

## Author Contributions

K.L.A.: Data curation, formal analysis, investigation, methodology, writing original draft, writing review – editing. N.F.: Data curation, Formal analysis, Investigation, Writing original draft, Writing review – editing. J.W.: Data curation, Formal analysis, Writing review – editing. P.S.: Conceptualization, Data curation, Formal analysis, Investigation, Methodology, Project administration, Supervision, Writing – review – editing.

## Funding

The authors have nothing to report.

## Ethics Statement

In compliance with Danish regulations on questionnaire‐based research, ethical approval was not deemed necessary for this study. All data were collected anonymously and all participants provided written consent for the use of their responses.

## Conflicts of Interest

The authors declare no conflicts of interest.

## Supporting information


**Table S1:** Prompt template employed with Chat GPT‐4o for the semantic clustering analysis.

## Data Availability

The data that support the findings of this study are available from the corresponding author upon reasonable request.

## References

[1] A. Donabedian , “Evaluating the Quality of Medical Care,” Milbank Quarterly 83 (2005): 691–729. 10.1111/j.1468-0009.2005.00397.x PMC2690293 16279964

[2] World Health Organization (WHO) , “Conceptual Framework for the International Classification for Patient Safety Final Technical Report,” Glossary of Patient Safety Concepts and References (2009): 3–101.

[3] A. Scott , W. Egner , D. J. Gawkrodger , et al., “The National Survey of Adverse Reactions to Dental Materials in the UK: A Preliminary Study by the UK Adverse Reactions Reporting Project,” British Dental Journal 196 (2004): 471–477. 10.1038/sj.bdj.4811176 15105862

[4] N. Ferlias , A. Michelotti , and P. Stoustrup , “Patient Safety in Orthodontic Care: A Scoping Literature Review With Proposal for Terminology and Future Research Agenda,” BMC Oral Health 24 (2024): 1–13. 10.1186/s12903-024-04375-7 PMC11184803 38890596

[5] P. Maramaldi , M. F. Walji , J. White , et al., “How Dental Team Members Describe Adverse Events,” Journal of the American Dental Association 147 (2016): 803–811. 10.1016/j.adaj.2016.04.015 PMC5045777 27269376

[6] E. S. Williams , L. B. Manwell , T. R. Konrad , and M. Linzer , “The Relationship of Organizational Culture, Stress, Satisfaction, and Burnout With Physician‐Reported Error and Suboptimal Patient Care: Results From the MEMO Study,” Health Care Management Review 32 (2007): 203–212. 10.1097/01.HMR.0000281626.28363.59 17666991

[7] N. Ferlias , H. Nielsen , E. Andersen , and P. Stoustrup , “Lessons Learnt on Patient Safety in Dentistry Through a 5‐Year Nationwide Database Study on Iatrogenic Harm,” Scientific Reports 14 (2024): 11436. 10.1038/s41598-024-62107-x PMC11102909 38763944

[8] J. Knights , L. Young , G. Humphris , T. Newton , and UK Dental Team Mental Health Research and Implementation Group , “Burnout and Depression in the UK Dental Workforce: Findings From a Cross‐Sectional Survey,” British Dental Journal 239 (2025): 189–196. 10.1038/s41415-025-8605-7 PMC12334352 40781428

[9] G. Humphris , J. Knights , L. Beaton , et al., “Exploring the Effect of the COVID‐19 Pandemic on the Dental Team: Preparedness, Psychological Impacts and Emotional Reactions,” Frontiers in Oral Health 2 (2021): 669752. 10.3389/froh.2021.669752 PMC8757713 35048012

[10] A. W. Wu , “Medical Error: The Second Victim. The Doctor Who Makes the Mistake Needs Help Too,” BMJ 320 (2000): 726–727. 10.1136/bmj.320.7237.726 PMC1117748 10720336

[11] A. M. Fahrenkopf , T. C. Sectish , L. K. Barger , et al., “Rates of Medication Errors Among Depressed and Burnt Out Residents: Prospective Cohort Study,” BMJ 336 (2008): 488–491. 10.1136/bmj.39469.763218.BE PMC2258399 18258931

[12] T. D. Shanafelt , C. M. Balch , G. Bechamps , et al., “Burnout and Medical Errors Among American Surgeons,” Annals of Surgery 251 (2010): 995–1000. 10.1097/SLA.0b013e3181bfdab3 19934755

[13] D. Seys , A. W. Wu , E. V. Gerven , et al., “Health Care Professionals as Second Victims After Adverse Events: A Systematic Review,” Evaluation & the Health Professions 36 (2013): 135–162. 10.1177/0163278712458918 22976126

[14] J. J. Mira , E. Potura , K. Schrøder , et al., “Standardizing Second Victim Support: Development of the RESCUE Certification Framework for Health Care Institutions,” BMC Health Services Research 25 (2025): 1539. 10.1186/s12913-025-13741-2 PMC12659461 41310735

[15] J. D. Burlison , S. D. Scott , E. K. Browne , S. G. Thompson , and J. M. Hoffman , “The Second Victim Experience and Support Tool: Validation of an Organizational Resource for Assessing Second Victim Effects and the Quality of Support Resources,” Journal of Patient Safety 13 (2017): 93–102. 10.1097/PTS.0000000000000129 PMC4342309 25162208

[16] N. Ferlias , A. Grigoroudi , J. Woolley , et al., “Patient Harm From Orthodontic Care (Part I): Types and Frequency of Patient‐Reported Harms From a National Database,” Orthodontics & Craniofacial Research (2026), 10.1111/ocr.70168. PMC13752659 42528037

[17] A. P. Sims , T. J. Roberts‐Harry , and D. P. Roberts‐Harry , “The Incidence and Prevention of Ocular Injuries in Orthodontic Practice,” British Journal of Orthodontics 20 (1993): 339–343. 10.1179/bjo.20.4.339 8286303

[18] E. Kalenderian , J. H. Lee , E. M. Obadan‐Udoh , A. Yansane , J. M. White , and M. F. Walji , “Development of an Inventory of Dental Harms: Methods and Rationale,” Journal of Patient Safety 18 (2022): 559–564. 10.1097/PTS.0000000000001033 PMC9391256 35771964

[19] S. Thusu , S. Panesar , and R. Bedi , “Patient Safety in Dentistry—State of Play as Revealed by a National Database of Errors,” British Dental Journal 213 (2012): E3. 10.1038/sj.bdj.2012.669 22878337

[20] E. Ensaldo‐Carrasco , M. F. Suarez‐Ortegon , A. Carson‐Stevens , et al., “Patient Safety Incidents and Adverse Events in Ambulatory Dental Care: A Systematic Scoping Review,” Journal of Patient Safety 00 (2016): 1–11. 10.1097/PTS.0000000000000316 27611771

[21] I. M. Busch , F. Moretti , M. Purgato , C. Barbui , A. W. Wu , and M. Rimondini , “Psychological and Psychosomatic Symptoms of Second Victims of Adverse Events: A Systematic Review and Meta‐Analysis,” Journal of Patient Safety 16 (2020): E61–E74. 10.1097/PTS.0000000000000589 PMC7386870 30921046

[22] M. A. Lane , B. M. Newman , M. Z. Taylor , et al., “Supporting Clinicians After Adverse Events: Development of a Clinician Peer Support Program,” Journal of Patient Safety 14 (2018): e56–e60. 10.1097/PTS.0000000000000508 PMC6102069 29878948

[23] L. New and T. Lambeth , “Second‐Victim Phenomenon,” Nursing Clinics of North America 59 (2024): 141–152. 10.1016/j.cnur.2023.11.011 38272580

[24] S. D. Scott , L. E. Hirschinger , K. R. Cox , M. McCoig , J. Brandt , and L. W. Hall , “The Natural History of Recovery for the Healthcare Provider ‘Second Victim’ After Adverse Patient Events,” Quality & Safety in Health Care 18 (2009): 325–330. 10.1136/qshc.2009.032870 19812092

[25] R. R. Quillivan , J. D. Burlison , E. K. Browne , S. D. Scott , and J. M. Hoffman , “Patient Safety Culture and the Second Victim Phenomenon: Connecting Culture to Staff Distress in Nurses,” Joint Commission Journal on Quality and Patient Safety 42 (2016): 377–384. 10.1016/s1553-7250(16)42053-2 PMC5333492 27456420

[26] E. Bailey and M. Dungarwalla , “Patient Safety in Dentistry ‐ a Decade in the Making,” British Dental Journal 238 (2025): 814–821. 10.1038/s41415-025-8384-1 PMC12101969 40410484

[27] D. Kahneman , Thinking, Fast and Slow (Farrar, Straus and Giroux, 2011), 1–15.

[28] G. Saposnik , D. Redelmeier , C. C. Ruff , and P. N. Tobler , “Cognitive Biases Associated With Medical Decisions: A Systematic Review,” BMC Medical Informatics and Decision Making 16 (2016): 1–14. 10.1186/s12911-016-0377-1 PMC5093937 27809908

[29] A. C. Edmondson , M. Higgins , S. Singer , and J. Weiner , “Understanding Psychological Safety in Health Care and Education Organizations: A Comparative Perspective,” Research in Human Development 13 (2016): 65–83.

[30] C. P. West , M. M. Huschka , P. J. Novotny , et al., “Association of Perceived Medical Errors With Resident Distress and Empathy: A Prospective Longitudinal Study,” JAMA 296 (2006): 1071–1078. 10.1001/jama.296.9.1071 16954486

[31] K. Schrøder , T. Bovil , J. S. Jørgensen , et al., “Evaluation of the Buddy Study, a Peer Support Program for Second Victims in Healthcare: A Survey in Two Danish Hospital Departments,” BMC Health Services Research 22, no. 1 (2022): 566, 10.1186/s12913-022-07973-9. PMC9043887 35477365

[32] World Health Organization , “WHO Patient Safety Research: Better Knowledge for Safer Care,” (2009).

[33] O. Tokede , M. Walji , R. Ramoni , et al., “Quantifying Dental Office–Originating Adverse Events: The Dental Practice Study Methods,” Journal of Patient Safety 17 (2021): e1080–e1087. 10.1097/PTS.0000000000000444 PMC10941983 29206706

[34] A. E. High and S. Forest , “Peer Support Targeting the Second Victim Phenomenon: Implementation and Outcomes,” Cureus 17, no. 2 (2025): e78854, 10.7759/cureus.78854. PMC11821368 39949643

[35] T. S. K. Ong , C. N. Goh , E. K. Y. E. Tan , et al., “Second Victim Syndrome Among Healthcare Professionals: A Systematic Review of Interventions and Outcomes,” Journal of Healthcare Leadership 17 (2025): 225–239. 10.2147/JHL.S526565 PMC12145115 40485771

