# Supplementary material for: Patient Harm From Orthodontic Care (Part III): The Care Provider Perspective and the Second Victim Phenomenon
Source: Orthod Craniofac Res. 2026 Oct 11;29(6):1172–82. doi: 10.1111/ocr.70195 (PMC13752633; doi:10.1111/ocr.70195)
Supplement: Supplementary file 1 [file OCR-29-1172-s001.docx]

**Supplementary Table 1**: Prompt template employed with Chat GPT-4o for the semantic clustering analysis.
